# Laser Acupuncture Therapy in Patients with Treatment-Resistant Temporomandibular Disorders

**DOI:** 10.1371/journal.pone.0110528

**Published:** 2014-10-17

**Authors:** Wen-Long Hu, Chih-Hao Chang, Yu-Chiang Hung, Ying-Jung Tseng, I-Ling Hung, Sheng-Feng Hsu

**Affiliations:** 1 Department of Chinese Medicine, Kaohsiung Chang Gung Memorial Hospital and Chang Gung University College of Medicine, Kaohsiung, Taiwan; 2 Kaohsiung Medical University College of Medicine, Kaohsiung, Taiwan; 3 Fooyin University College of Nursing, Kaohsiung, Taiwan; 4 Division of Chinese Medicine, Kaohsiung Municipal Chinese Medical Hospital, Kaohsiung, Taiwan; 5 School of Chinese Medicine for Post Baccalaureate, I-Shou University, Kaohsiung, Taiwan; 6 Graduate Institute of Acupuncture Science, China Medical University, Taichung, Taiwan; 7 Department of Chinese Medicine, China Medical University Hospital, Taipei Branch, Taipei, Taiwan; Stavanger University Hospital, Norway

## Abstract

**Objective:**

To investigate the clinical effects of laser acupuncture therapy for temporomandibular disorders (TMD) after ineffective previous treatments.

**Methods:**

A retrospective observational study was conducted in 29 treatment-resistant TMD patients (25 women, 4 men; age range, 17–67 years). Subjects were treated 3 times per week for 4 weeks with the Handylaser Trion (GaAlAs laser diode, 810 nm, 150 mW, pulsed waves), which delivered 0.375 J of energy (5 s) to ST7, ST6, and LI4 and 3 J (40 s) to each Ashi point, 7.5–26.25 J/cm^2^ in total. The visual analog scale (VAS) and maximal mouth opening (MMO) were evaluated before and after treatment.

**Results:**

VAS analysis showed that the patients were free of pain at rest (endpoint) after 5.90±6.08 sessions of laser acupuncture for acute TMD and after 16.21±17.98 sessions for chronic TMD. The VAS score on palpation of the temporomandibular joint reduced to 0.30±0.67 for patients with acute TMD (*p* = 0.005) and to 0.47±0.84 for those with chronic TMD (*p*<0.001). The MMO significantly increased in patients with acute TMD (7.80±5.43 mm, *p* = 0.008) and in patients with chronic TMD (15.58±7.87 mm, *p*<0.001).

**Conclusions:**

Our study shows that laser acupuncture therapy improves the symptoms of treatment-resistant TMD. Further studies with a more appropriate design, involving long-term follow-up examinations in a larger patient sample, are needed to evaluate its efficacy.

## Introduction

Temporomandibular disorder (TMD) is a collective term traditionally used to describe multiple disorders, including intracapsular disorders, true abnormalities of the temporomandibular joint (TMJ), and muscular disorders or myofascial pain dysfunction (MPD) syndrome [1]. TMD is a major cause of nondental pain in the orofacial region. In the adult non-patient population, approximately 33% reported at least one TMD symptom, and clinical findings revealed at least one TMD sign in 40%–75% of the population [2]. It can be a very painful condition, leading to significant deterioration in the patient’s quality of life. The primary symptoms associated with TMD include facial muscle pain, preauricular (TMJ) pain, TMJ sounds (jaw clicking, popping, catching, and locking), limited mouth opening, and increased pain associated with chewing. The secondary symptoms are earache, headache, and neck ache [3]. Bruxism, teeth clenching, and chronic gum chewing are important factors influencing the production of pain in the masseter and temporalis muscles. Arthritis and degenerative changes in the TMJ, loss of teeth, ill-fitting dentures or lack of dentures, and other dental conditions can lead to TMD or MPD, which manifest as facial and masticatory muscle pain [4].

The roles of electrophysical modalities and surgery in the management of TMD have not been fully elucidated [5]. Initial conservative therapy is based on 3 general approaches: patient education, pharmacologic therapy, and physical therapy. However, patients with chronic TMD usually need a multidisciplinary approach involving a team of therapists, including a dentist, psychologist, physical therapist, and even a chronic pain physician [3].

Among the therapeutic procedures, low-level laser therapy (LLLT) has recently been proposed to reduce pain intensity and improve maximal mouth opening (MMO) in both acute and chronic TMD patients who had received no previous TMD treatment (e.g., surgical treatment, occlusal splint, or LLLT) [6]. A systematic review reported that LLLT is probably more effective for the treatment of TMJ disorders, and less effective for the treatment of masticatory muscle disorders [7]. One systematic review and meta-analysis showed that there is limited evidence for symptomatic treatment of TMD with acupuncture [8]. Another study suggested that acupuncture is a reasonable adjunctive treatment for producing a short-term analgesic effect in patients with painful TMD symptoms [9]. Furthermore, laser acupuncture therapy (LAT) has been proposed as an effective treatment modality because it alleviates the chronic pain associated with TMD without any previous treatment [10]. However, the effect of LAT on TMD symptoms in patients in whom previous treatments were unsuccessful has not been investigated. Therefore, in this study, we investigated the effect of LAT in such patients.

## Materials and Methods

### Patients

**Figure 1:**
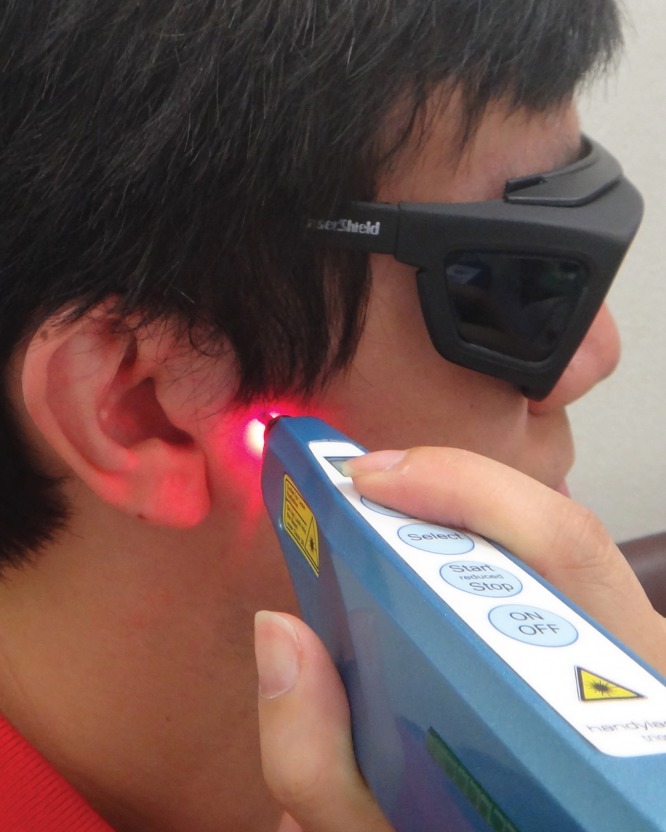
Laser acupuncture performed using the Handylaser Trion at ST7.

The study was approved by the Human Ethics Committee of our hospital (Chang Gung Medical Foundation Institutional Review Board, Permit No 102-1932B). This is a retrospective observational study in general practice. The patient's verbal informed consent for treatment was recorded on the medical chart. Our ethics committee approved the verbal consent procedure. Furthermore, the individual in this manuscript (Figure 1) has given written informed consent to publish these case details. Patients who visited our outpatient acupuncture clinic between January 2009 and December 2011 were recruited for this study. In total, 25 women and 4 men (age range, 17–67 years; mean ± standard deviation, 42.48±14.77) with treatment-resistant TMD were enrolled. They were diagnosed with TMD (cardinal signs: limitation of jaw opening or function, pain with jaw opening or function, and joint sounds [11]) and treated by dentists, and referred to us because of unsuccessful previous treatments such as advice and counseling regarding their diet, cold/hot packs, administration or topical use of nonsteroidal anti-inflammatory drugs, and/or an occlusal appliance. Seven patients had bilateral TMD, and the rest had unilateral TMD. The duration of symptoms from the onset of disease ranged 1–144 months (17.45±31.64). In 10 patients, the symptoms lasted for <6 months (acute TMD), and in the remaining patients, they lasted for ≥6 months (chronic TMD) [12]. Patients were instructed not to resort to self-medication.

### Laser acupuncture therapy

The patients were treated 3 times per week for 4 weeks (recommendation based on clinical experience; treatment could be extended if necessary) with the Handylaser Trion (Table 1, RJ-Laser, Germany) by an experienced physician (acupuncture specialist with total acupuncture experience of 25 years). Protective goggles were used by both the operator and the patient to inhibit visual perception during LAT. The laser was used to deliver 0.375 J of energy in pulsed waves to each of the following traditional acupoints for 5 s sequentially: ST7 (Xiaguan, B2: 1199 Hz) (Figure 1), ST6 (Jiache, B2: 1199 Hz), and LI4 (Hegu, B3: 2398 Hz). In addition, the laser was applied to the Ashi points on and near the TMJs and/or masseter muscles (2–4 points) for 40 s each, delivering 3 J of energy in pulsed wave (NC′: 1168 Hz), 7.5–26.25 J/cm^2^ in total.

**Table 1:** The set parameters of the Handylaser Trion used to perform laser acupuncture in this study.

Laser medium	GaAlAs laser diode
Wavelength	810 nm
Output power - maximum	150 mW
Probe aperture	0.03 cm^2^
Power density	5 W/cm^2^
Time	5 s/acupuncture point; 40 s/Ashi point
Frequencies	Bahr (B1: 599.5 Hz, B2: 1199 Hz, B3: 2398 Hz, B4: 4796 Hz, B5: 9592 Hz, B6: 19184 Hz, B7: 38360 Hz) Nogier (NA′: 292 Hz, NB′: 584 Hz, NC′: 1168 Hz, ND′: 2336 Hz, NE′: 4672 Hz, NF′: 9344 Hz, NG′: 18688 Hz)
Type of application	Contact
Treatment dose	7.5–26.25 J/cm^2^ in total

### Outcome measures and data analysis

A 10-cm visual analog scale (0–10 points, least to greatest pain intensity, VAS) was used to measure the pain intensity at rest (spontaneous, VASS) and upon digital palpation (0.5 kg of pressure [13] applied by Dr. WLH) of the TMJ (VASP). To measure MMO, patients were asked to open their mouths as wide as possible without assistance. The interincisal distance in mm was recorded using a ruler. SPSS Statistics 17.0 (SPSS Inc., Chicago, USA) was used to analyze the data. The Wilcoxon signed-rank test was used to compare the VAS and MMO results before treatment with those obtained after LAT. All *p*-values were 2-tailed, and the alpha level of significance was set at 0.05.

## Results

After 12.66±15.64 therapeutic sessions, the patients experienced pain relief and improvement in MMO. After 5.90±6.08 therapeutic sessions, the acute TMD patients reported that they were free of pain at rest (endpoint); in contrast, chronic TMD patients required 16.21±17.98 therapeutic sessions before they could report a similar outcome (Table 2). After 8.73±7.44 therapeutic sessions, the patients without disc displacement reported that they were free of pain at rest; in contrast, the patients with disc displacement required 15.06±18.82 therapeutic sessions before they could report a similar outcome. No negative side effects were reported during or after LAT.

**Table 2:** Patient demographics before and after LAT.

No.	Gender	Age	Site	Course (M)	VASSB	VASSA	VASPB	VASPA	MMOB	MMOA	Sessions	Disc displacement
1	M	45	R	1	4.5	0.0	6.5	0.0	40.0	41.0	1	None
2	F	26	L	1	4.0	0.0	6.0	0.0	38.0	45.0	3	R
3	F	57	R	1	4.0	0.0	6.0	0.0	32.0	42.0	14	R
4	F	48	R	1	7.0	0.0	8.0	0.0	32.0	43.0	18	None
5	F	54	L	1	7.0	0.0	8.0	0.0	33.0	42.0	6	None
6	F	58	R	1	4.5	0.0	6.5	0.0	34.0	40.0	1	R
7	F	42	L	1	8.0	0.0	8.0	2.0	39.0	39.0	10	R
8	F	39	R	2	7.0	0.0	8.0	0.0	30.0	40.0	2	None
9	M	23	B	3	4.0	0.0	6.0	0.0	35.0	40.0	1	None
10	M	58	R	4	7.0	0.0	8.0	1.0	16.0	35.0	3	None
11	M	58	L	6	9.0	0.0	9.0	0.0	16.0	38.0	72	NR
12	F	44	B	6	4.0	0.0	6.0	0.0	30.0	41.0	54	R
13	F	40	R	6	4.0	0.0	6.0	0.0	30.0	40.0	2	R
14	F	40	B	6	7.0	0.0	8.0	2.0	16.0	34.0	6	NR
15	F	26	R	6	7.0	0.0	8.0	0.0	16.0	42.0	19	NR
16	F	17	L	6	4.0	0.0	6.0	0.0	32.0	36.0	19	R
17	F	58	L	6	7.0	0.0	8.0	0.0	32.0	42.0	20	None
18	F	23	B	10	7.0	0.0	8.0	1.0	35.0	39.0	1	R
19	F	62	B	12	7.0	0.0	8.0	1.0	16.0	38.0	4	NR
20	F	39	L	12	7.0	0.0	8.0	0.0	33.0	41.0	6	None
21	F	38	R	12	7.0	0.0	8.0	3.0	16.0	40.0	13	NR
22	F	17	L	12	7.0	0.0	8.0	0.0	16.0	40.0	16	NR
23	F	21	L	12	7.0	0.0	8.0	0.0	16.0	40.0	19	NR
24	F	54	B	18	7.0	0.0	8.0	0.0	33.0	41.0	6	None
25	F	29	L	24	7.0	0.0	8.0	1.0	16.0	35.0	4	R
26	F	45	R	36	4.0	0.0	6.0	0.0	33.0	41.0	4	R
27	F	43	R	60	7.0	0.0	8.0	1.0	16.0	38.0	10	NR
28	F	61	L	96	8.0	0.0	8.0	0.0	16.0	40.0	16	None
29	F	67	B	144	7.0	0.0	8.0	0.0	33.0	41.0	17	None

LAT: laser acupuncture therapy; MMOA: Maximal mouth opening after LAT; MMOB: Maximal mouth opening before LAT; NR: without reduction; R: with reduction; VASPA: Visual analog scale upon palpation after LAT; VASPB: Visual analog scale upon palpation before LAT; VASSA: Visual analog scale, spontaneous (at rest), after LAT; VASSB: Visual analog scale, spontaneous (at rest), before LAT.

### Reduction in pain intensity

Before treatment, all 29 patients reported a VASS score of 6.24±1.52, and VASP score of 7.45±0.94. The mean VASS score before LAT was 5.70±1.62 for acute TMD patients and 6.53±1.43 for chronic TMD patients. The mean VASP score before LAT was 7.10±0.97 for acute TMD patients and 7.63±0.90 for chronic TMD patients.

After treatment, all 29 patients reported no pain at rest (Wilcoxon test, z = –4.828, *p*<0.001), and a VASP score of 0.41±0.78 upon palpation of the TMJ (Wilcoxon test, z = –4.753, *p*<0.001). The mean VASP score was 0.30±0.67 for acute TMD patients (Wilcoxon test, z = –2.831, *p* = 0.005) and 0.47±0.84 for chronic TMD patients (Wilcoxon test, z = –3.868, *p*<0.001; Table 3).

**Table 3:** Effect of LAT on treatment-resistant TMD.

Variable	VASS	VASP	MMO
Total, *n* = 29			
Before LAT	6.24±1.52	7.45±0.94	26.90±8.96
After LAT	0	0.41±0.78	39.79±2.47
A–B	−6.24±1.52	−7.10±1.04	12.90±7.97
Z	−4.828	−4.753	−4.628
*P* value	0.000	0.000	0.000
Acute TMD, *n* = 10			
Before LAT	5.70±1.62	7.10±0.97	32.90±6.79
After LAT	0	0.30±0.67	40.70±2.67
A–B	−5.70±1.62	−6.80±0.89	7.80±5.43
Z	−2.831	−2.831	−2.668
*P* value	0.005	0.005	0.008
Chronic TMD, *n* = 19			
Before LAT	6.53±1.43	7.63±0.90	23.74±8.44
After LAT	0	0.47±0.84	39.32±2.29
A–B	−6.53±1.43	−7.26±1.10	15.58±7.87
Z	−3.976	−3.868	−3.833
*P* value	0.000	0.000	0.000

Wilcoxon signed-rank test; A–B: after–before LAT; LAT: laser acupuncture therapy; MMO: Maximal mouth opening; TMD: temporomandibular disorder; VASP: Visual analog scale upon palpation; VASS: Visual analog scale, spontaneous (at rest).

### Improvement in maximal mouth opening

Before treatment, the MMO for all 29 patients with treatment-resistant TMD was 26.90±8.96 mm. The MMO for patients with acute TMD was 32.90±6.79 mm, whereas the MMO for patients with chronic TMD was 23.74±8.44 mm.

After treatment, the MMO for all 29 patients with treatment-resistant TMD was 39.79±2.47 mm (Wilcoxon test, z = –4.628, *p*<0.001). The MMO for patients with acute TMD was 40.70±2.67 mm (Wilcoxon test, z = –2.668, *p* = 0.008), whereas the MMO for patients with chronic TMD was 39.32±2.29 mm (Wilcoxon test, z = –3.833, *p*<0.001; Table 3).

## Discussion

The suppression of cortical responses and brainstem reflexes is elicited by a predominantly nociceptive input in TMD patients, which suggests that chronic craniofacial pain in TMD patients may be associated with dysfunction of the trigeminal nociceptive system [14]. Increased pain intensity in patients with MPD-TMD at rest has been associated with increased gray matter in the rostral anterior cingulate cortex and posterior cingulate. In the same study, pain intensity with palpation was associated with decreased gray matter in the pons, corresponding to the trigeminal sensory nuclei. Longer pain duration was associated with greater gray matter in the posterior cingulate, hippocampus, midbrain, and cerebellum. The pattern of gray matter abnormalities found in MPD-TMD individuals suggests the involvement of trigeminal and limbic system dysregulation, as well as potential somatotopic reorganization in the putamen, thalamus, and somatosensory cortex [15].

According to the theory of traditional Chinese medicine (TCM), pain results from blood stasis due to *qi* stagnation (a pathological change in which a long-standing or severe stagnation of *qi* impedes blood flow, a condition characterized by the coexistence of *qi* stagnation and blood stasis) [16], [17]. We selected the acupoints on both Yangming meridians based on the principle of “places where meridian passed, treatments thereby can be reached” [18]. According to Manfred Reininger et al., frequencies can be applied to acupuncture points to improve the meridian energy [19]. The ST7 (B2: 1199 Hz used for central tissue layer), ST6 (B2: 1199 Hz), and LI4 (B3: 2398 Hz used for surface tissue structures [20]) points are involved in freeing the meridians and collaterals, and moving *qi* to relieve pain. The combined use of these acupoints is therefore effective in activating blood and moving *qi*, anti-inflammation, and analgesia. In addition to stimulating Ashi points (NC′: 1168 Hz used for circulation, energy transfer, and locomotor disorders [20]) on and near the TMJ and/or masseter muscle, the flow of *qi* and blood in the body is realigned to restore internal homeostasis and resolve the symptoms of disease.

LAT is a noninvasive technique involving the stimulation of traditional acupoints, with low-intensity, non-thermal laser irradiation. The clinical application of LAT is widespread, although its mechanism is not well understood. LLLT has biologic effects, such as increased pain tolerance, due to changes in the potency of the cellular membrane; vasodilatation; reduction of edema; increase in intracellular metabolism; and acceleration of wound healing [21]. The biomodulatory effect of LLLT improves local microcirculation and oxygen supply to hypoxic cells in the painful areas. Simultaneously, tissue asphyxia is reduced to a minimum and collected waste products are removed. The laser-induced normalization of microcirculation interrupts the vicious cycle that originates, develops, and maintains pain; in addition, it restores the normal physiological condition of the tissue [22], [23]. Research has shown that LLLT can modulate inflammation by reducing the levels of biochemical markers (prostaglandin E2, messenger ribonucleic acid cyclooxygenase-2, interleukine-1β, and tumor necrotizing factor-α), neutrophil influx, oxidative stress, edema, and hemorrhage in a dose-dependent manner [24].

Analgesia induced by LLLT is mediated by peripheral opioid receptors [25]. Laser acupuncture is a modality resulting from scientific exploration of TCM [10]. Acupuncture has both local and distant analgesic effects that may be mediated by different mechanisms. Various central opioid receptors are important in mediating the analgesic effect induced by acupuncture-related techniques of different frequencies [26]. In comparison with needle-based acupuncture for achieving *qi*, LAT is not associated with somatosensation and has the advantage of being noninvasive and aseptic. Moreover, LAT is painless and safe because no heat is generated during the procedure. It is also more effective and requires less time than needle-based acupuncture [19], [27]. Although Melis et al. hypothesized that when structural or functional problems are present (for example, a displaced disc), the effects of the laser beam cannot sufficiently alleviate the symptoms until the main cause is addressed [7]. Our patients with disc displacement reported that they were free of pain at rest after 15.06±18.82 therapeutic sessions. LAT might act on the synovia and stimulate cellular energy processes [28]. The results of our study are similar to those of Ferreira et al.’s study [10], in which equivalent methodology was used. Furthermore, no side effects or complications resulting from LAT were observed in our study.

TMDs occur disproportionately in women of childbearing age in a female to male ratio of between 4:1 and 6:1; the prevalence decreases significantly in men and women above 55 years of age [11]. We also noted this disproportion in our treatment-resistant TMD patients. In this study, although most patients reported a drastic reduction in pain intensity after LAT, 2 patients with acute TMD reported a VASP score of 1–2 after treatment, and 6 patients with chronic TMD reported a VASP score of 1–3. With regard to the etiological multifactorial aspects of TMD, LR3 (Taichong) might be used in combination with LI4 to alleviate conditions of stress, depression, and anxiety, as well as pain, by promoting the free flow of energy and emotions [10]. Tunér and Hode [29] reported that there is a methodological association with local laser action in clinical practice and in some studies involving LAT. It is known that the laser in the irradiated region is also capable of promoting punctiform analgesia, particularly when the equipment is specifically adjusted [30]. Future studies are needed to verify the effect of the local laser beyond the acupoints, the selection of the acupoints; treatment intervals; therapeutic sessions; and optimal laser parameters, including wavelength, dose, and intensity, necessary to maximize the physiological benefit and cost effectiveness of LAT for treatment-resistant TMD.

## Conclusions

We found that TMD symptoms improved with LAT. Given the relative scarcity of research on this topic, long-term follow-up of these cases and further studies with a larger patient sample and an appropriate design are needed to elucidate the efficacy of LAT in treatment-resistant TMD patients.
